# Ti_3_C_2_T_x_ MXene-Polymeric Strain Sensor with Huge Gauge Factor for Body Movement Detection

**DOI:** 10.3390/mi13081302

**Published:** 2022-08-12

**Authors:** Wei Xian Rebecca Leong, Adel Mohammed Al-Dhahebi, Mohamad Radzi Ahmad, Mohamed Shuaib Mohamed Saheed

**Affiliations:** 1 Department of Fundamental and Applied Sciences, Universiti Teknologi PETRONAS, Seri Iskandar 32610, Perak, Malaysia; 2 Centre of Innovative Nanostructure & Nanodevices (COINN), Universiti Teknologi PETRONAS, Seri Iskandar 32610, Perak, Malaysia; 3 Department of Mechanical Engineering, Universiti Teknologi PETRONAS, Seri Iskandar 32610, Perak, Malaysia; 4 Department of Electrical & Electronics Engineering, Universiti Teknologi PETRONAS, Seri Iskandar 32610, Perak, Malaysia

**Keywords:** Ti_3_C_2_T_x_-MXene, polypyrrole, strain sensing, torsion

## Abstract

In this work, a composite strain sensor is fabricated by synthesizing MXene and deposition of polypyrrole on top of the flexible electrospun PVDF nanofibers. The fabricated sensor exhibits a conductive network constructed with MXene and polypyrrole of microcracks network structure, demonstrating its strain sensing properties. The presence of these microcracks serves as mechanical weak points, which leads to sensitivity enhancement, while the electrospun fiber substrate act as a cushion for strain loading under large deformations. The as-prepared MXene@Polypyrrole PVDF sensor has a gauge factor range of 78–355 with a sensing range between 0–100%. Besides strain deformations, the sensor can operate in torsional deformation and human motion, indicating the sensor’s potential as a wearable health monitoring device.

## 1. Introduction

MXene, a new class of 2D material, has been researched broadly recently. This material class comprises layers of transition metal/carbides, nitrides, or carbonitrides [1]. MXene is stripped from the ternary ceramic structure carbide and nitrides with the formula M_n+1_AX_n_, n = 1,2, or 3, where M is the transition metal (Ti, V, Cr, Mo, Nb, etc.), A is mainly group 13 and 14 elements (Si, Al, Ge or Sn) and X is carbon, nitrogen or both [2,3,4]. The combination of ceramic and metallic behaviors makes the MAX phase distinctive. MAX phase exhibits high hardness, low density, and resistance to corrosion. Besides, its high thermal and electrical conductivity properties are near to metallic materials [5]. Among the different classes of MXene, Ti_3_ C_2_ T_x_ is highly investigated. A high selectivity etching agent is crucial to etch the Al atom layer from the Ti_3_AlC_2_ accurately. MXene is synthesized from its parental MAX precursor phase using the topochemical selective etching process. The principle of this method involves the use of chemical etchant (hydrofluoric acid (HF) or hydrochloric acid/lithium fluoride (HCL/LiF)) to etch or substitute (delaminate) the strong M-A bonds (in the MAX phase) with weaker hydroxyl (OH), oxygen (O), fluorine bonds. The resultant materials typically possess stoichiometry structure of M_n+1_X_n_T_x_ layers (called MXene) in which the T_x_ describes the surface functional groups (OH, O, and F) [1]. These surface terminations render MXene hydrophilic functionality and processability compared to other 2D nanomaterials such as graphene and transition-metal dichalcogenides (TMDs). As a result, MXene based materials have recently captivated the interests of scientists and engineers across various disciplines not only in biomedical applications but also in wearable, soft electronics and energy storage devices [5].

Strain sensors or conventionally known as strain gauge have been in the market for decades. The basic concept of a strain sensor is it transduces mechanical deformations into electrical signals. Flexible and stretchable strain sensor still focus on piezoresistive and capacitive type due to their simple read-out system and high flexibility and stretchability. Common intrinsic stretchability of strain sensor is incorporated with conductive materials such as carbon black, graphene, carbon nanotubes (CNTs) and metal nanostructures (e.g., AgNWs) into stretchable elastomeric matrix. Stretchable conductors with huge difference in electrical properties during deformation are commonly utilized in stretchable devices. However, there are several drawbacks associated with these nanostructured conductive materials such as low electrical conductivity (carbon black), irreversible cracks after deformation (graphene), poor purity causing large resistance (CNT) and relatively high cost fabrication process (AgNWs) [5].

Recently, MXene-based strain sensors performance outperformed previously reported hydrogel [6] and MoS_2_ [7]. They exhibit characteristics of high sensitivity, fast response, good stability, and excellent conformability. Due to MXene hydrophilic behavior has good interaction with polymeric matrixes, it is favorable in synthesis of hybrid composite materials. This composite can be processed through simple and low-cost methods into films, fibers and 3D structures attributed to easy fabrication methods such as electrospraying. Several polymer matrixes were reported, including polyvinyl alcohol (PVA) [8,9,10], poly(vinylidene fluoride), polyacrylamide (PAM) [11], polyethylene [12], polyurethane (PU) [13], polyfluorenes [14] and polydiallyldimethylammonium chloride (PDDA) [8] which were utilized as a base substrate for flexibility of the strain sensor. However, MXene incorporation affects the structure and crystallization rate of the polymer matrix which influences the overall performance of the device [12]. Among various conducting polymers, polypyrrole (PPy) is one of the most successful electrode materials due to its easy synthesis, low cost, and high conductivity [15]. In previous studies, conductive PPy/PU composites fabricated strain sensors with high stretchability but had relatively low sensitivity for a strain sensing application [16]. Recently, another group of researchers produced MXene/PVDF composite to demonstrate the excellent contribution of MXene to electrical performance [17]. To the best of our knowledge, there are no reports on incorporating MXene nanosheets with PPy on PVDF nanofiber in the application of strain sensing and human motion monitoring. Despite the excellent properties of polymer and MXene composite, there is still limited detailed study that discussed the changes in conductivity and structures of polymeric composites with MXene, crucial in utilizing for real-time applications such as in healthcare. 

Herein, we report a process using Ti_3_C_2_T_x_ MXene and polypyrrole as conductive material and electrospun polyvinylidene fluoride (PVDF) as the flexible substrate. The synthesized MXene powder was mixed in stoichiometric ratio with pyrrole monomer, then deposited on the electrospun PVDF substrate. The fabricated conductive flexible substrate is processed into sensor packaging for electromechanical characterization.

## 2. Materials and Methods

### 2.1. Preparation

#### 2.1.1. Synthesis of MXene

The MXene powder Ti_3_C_2_T_x_ was prepared using the chemical liquid etching method adopted from Alhabeb et al. [18]. The precursor material Ti_3_AlC_2_ (99 wt%, Luoyang Tongrun Info Technology Co., Ltd., Luoyang, China) was etched by the Al layer via 9 M HCl (37 wt%, Sigma–Aldrich, St. Louis, MO, USA) and 12M LiF (99 wt%, Sigma–Aldrich, St. Louis, USA). During the acid etching process, Ti_3_AlC_2_ powder was introduced into the acid mixture at a ratio of 1 g:10 mL under continuous stirring for 24 h at room temperature. The resulting mixture was washed with distilled water and centrifuged at 7500 rpm for 5 min until the pH was above 6. The precipitates were then collected by vacuum filtration through PVDF membrane and dried in a vacuum oven at 80 °C for 6 h. The dried residues are collected from the PVDF membrane for further characterization.

#### 2.1.2. Electrospun PVDF Fabrication

Electrospinning is a versatile and viable technology capable of manufacturing various nanofibrous assemblies from a variety of raw materials (polymers, metals, ceramics, etc.) with specialized features such as large surface area, interconnected and porous structures, easy functionalization and surface modification, and mechanical robustness [19]. The unique features of electrospun nanofibers make them appealing for the design of high-performance wearable and strain sensors, biomedical devices, energy storage and filters [20,21]. Electrospinning was used to fabricate electrospun PVDF nanofibers and discussed in great detail in our previous publications [22,23]. Briefly, electrospun PVDF solution was prepared by dissolving 20 wt.% in acetone/dimethylformamide (DMF) (2:3 *v*/*v*) and magnetically stirred at 60 °C for 5 h to dissolve the PVDF followed by stirring overnight at room temperature to obtain homogenous solution. The solution was loaded into a 10 mL plastic syringe with stainless steel needle (21 G) and used for electrospinning (ES: Model TL-Pro-BM) under spinning conditions of 2 mL/h flow rate, 15 cm as fiber receiving distance, and 18 kV electrical applied voltage. The electrospinning process was conducted at room temperature and with less than 30% relative humidity. The ejected nanofibers were collected using a grounded metallic collector that was covered with aluminum foil and revolving at 400 rpm. The electrospun nanofibers were dried in a drying oven at 60 °C for 12 h to remove the residual solvents. The electrospun PVDF nanofibers were then used for the preparation of the strain sensor as illustrated in the subsequent section. 

#### 2.1.3. Sensor Fabrication

**Figure 1:**
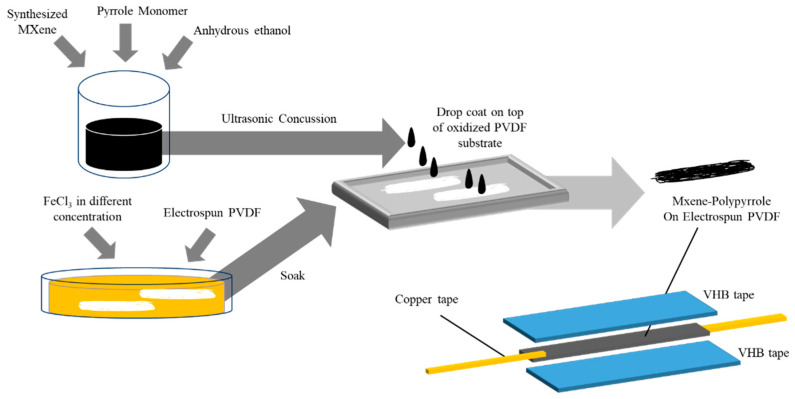
Schematic diagram of fabrication process of Mxene@Polypyrrole electrospun PVDF sensor.

First, FeCl_3_ solution with different concentrations was prepared ranged from 0.1 M to 0.5 M. Electrospun PVDF strips are cut into the desired length (5 × 1 cm^2^) [24] and soak in FeCl_3_ solution of different concentrations for 2 h. Pyrrole monomer and anhydrous ethanol were mixed in 1:1 ratio as pyrrole solution. 5 wt% of MXene in pyrrole solution was prepared and ultrasonicated for 30 min. The electrospun PVDF strip was then laid flat on Teflon plates with 5 wt% MXene-PPy solution drop coated on top of the PVDF strip. The process of reaction was allowed for 2 h and the PVDF strip was rinsed with DI water and ethanol. The conductive PVDF strip was then sandwiched between flexible VHB tape (4910F, 3M) and attached with copper tape at both ends of the conductive PVDF strip as indicated in Figure 1. Different sensing films denoted as Ti_3_C_2_T_x_/PPy_0.1_/PVDF, Ti_3_C_2_T_x_/PPy_0.2_/PVDF, Ti_3_C_2_T_x_/PPy_0.3_/PVDF, Ti_3_C_2_T_x_/PPy_0.4_/PVDF and Ti_3_C_2_T_x_/PPy_0.5_/PVDF are designed and fabricated, respectively. The subscript numbers represent the concentration of the FeCl_3_ used. 

### 2.2. Characterization

Characterization tools used were Field Emission Scanning Electron Microscope (FESEM, Supra 55VP, ZEISS, Jena, Germany), Transmission Electron Microscopy (TEM, HT7830, Hitachi, Tokyo, Japan), and X-ray Photoelectron Spectroscopy (XPS, K-Alpha, Thermo, Waltham, MA, USA). The X-ray diffraction spectrum of the samples was obtained on a powder X-ray diffractometer (Xpert3 Powder, Panalytical, Malvern, UK) using Cu Kα (λ = 1.5418) radiation as the light source. The 2θ angle was investigated for 5–80°. The FTIR spectra were recorded on Fourier-transform infrared spectroscopy (FTIR, Frontier, Perkin Elmer, Waltham, MA, USA). The electromechanical characterization of the strain sensors was measured with a static in-house build sensor holder and a digital multimeter (31944A, Keysight, Santa Rosa, CA, USA). All characterizations are performed in room temperature.

## 3. Material Characterization

A schematic illustration of the PVDF/Ti_3_C_2_T_x_ MXene@Polypyrrole strain sensor fabrication procedure is illustrated in Figure 1. First, a mixed solution of synthesized MXene (Ti_3_C_2_T_x_) nanosheets pyrrole monomer was prepared through an ultrasonic concussion to produce the conductive filler solution. The flexible substrate, electrospun PVDF nanofiber was prepared by soaking in initiator solution and conductive filler solution was coated on top of the flexible substrate producing a conductive flexible electrode. The conductive substrate was then packaged into a functional strain sensor. 

**Figure 2:**
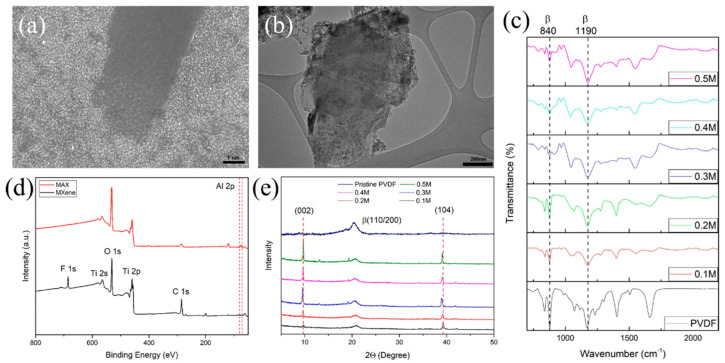
(**a**) FESEM images of Ti_3_C_2_T_x_ nanosheets (**b**)TEM images of Ti_3_C_2_T_x_ nanosheets (**c**) FTIR spectrum of various FeCl_3_ concentration (**d**) XPS spectrum of MAX phase and Ti_3_C_2_T_x_ nanosheets (**e**) XRD spectrum of electrospun PVDF and various FeCl_3_ concentration.

Figure 2a shows the FESEM image of MXene nanosheets, confirming the etching of Al and delamination of MXene layers. Additionally, in Figure 2b the TEM image of MXene nanosheets indicates MXene sheets are well exfoliated. The most intense XRD peak (002) at 9° confirmed the proper synthesis of MXene as shown in Figure 2e. The surface functionalization of MXene was analyzed with XPS, shown in Figure 2d. The XPS spectrum exhibit the presence of F, O, Ti, and C elements with the notable absence of the metallic Al. The binding energies of the core peak position of F, O, Ti, and C elements are 684.7, 532.4, 455.8, and 284.9 eV, respectively. The presence of O and F indicates the surface of MXene film is functionalized by –O, –OH, and –F groups which is consistent with previous reports [17]. Besides that, no significant peak observed between 70–80 eV represents the Al 2p peak of MAX phase, further confirming the successful synthesis of MXene.

The PVDF sheets employed in this study is an electrospun PVDF consisting of chemical structures of β-phase of the PVDF [22,23]. The spinning process consists of viscous PVDF precursor solution melt in applied electric field, causing uniaxial stretching due to the repulsion of the electrostatic force between surface charges. This develops the transition of α-phase crystallization into the β-phase crystallization of electrospun PVDF nanofiber [25]. The flexible electrospun PVDF nanofiber substrate will be decorated with MXene and polypyrrole, making the overall structure conductive. Polar functional groups such as –O, –OH, and –F groups are formed on the surface of MXene after chemical etching, leading to oxidation and decreasing the conductivity of MXene. Therefore, to improve the long-term stability of MXene is through modification by integrating PPy. The polymerization of PPy includes an initiator, FeCl_3_. The polymerization of pyrrole monomers can be controlled to form PPy on the surface of MXene and PVDF substrate. The deposition of PPy not only facilitates the assembly of MXene onto PVDF substrate but also improves the non-conductive electrospun PVDF nanofiber by varying the amount of initiator. The PVDF substrate act as a support network for the formation of polypyrrole along with Ti_3_C_2_T_x_ MXene nanosheets. The synergy of the composition harmoniously modifies the fibre with electrical properties. 

**Figure 3:**
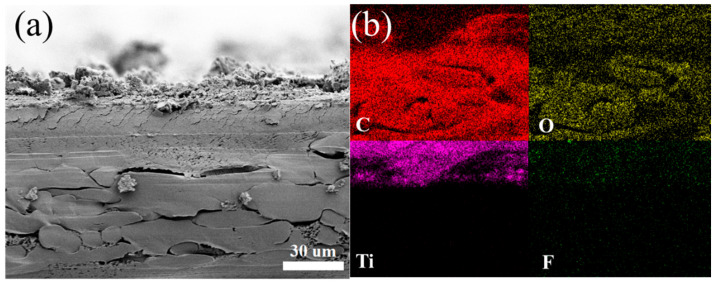
(**a**) Cross-sectional FESEM images of Ti_3_C_2_T_x_/PPy coated electrospun PVDF (**b**) EDS elemental mapping images of C, O, Ti, and F for the Ti_3_C_2_T_x_/PPy coated electrospun PVDF.

The surface morphology of the fabricated flexible PVDF substrate after the deposition of conductive filler was analyzed by FESEM, XRD, and FTIR analysis. Figure 2e shows the XRD analysis of PVDF substrate with different concentrations of the precursor solution. The strong prominent peak (002) and (104) planes at 9.6° and 38.7°, respectively, for all the fabricated samples obtained from the MXene nanosheets. As filler concentration increase, the β-characteristic (110/200 planes) become wider. The FTIR spectra are illustrated in Figure 2c. The intensity corresponding to the absorption bands at 840 and 1190 cm^−1^ of the β-crystal plane is reduced after increasing the concentrations of precursor solution in the PVDF, which also follows the observation in the XRD pattern. Figure 3a shows the cross-sectional FESEM image of Ti_3_C_2_T_x_/PPy coated electrospun PVDF, which clearly represents the deposited conductive layer is firmly attached without peeling or revealing the surface of the PVDF layer. Moreover, the uniform element C, O, Ti, and F distribution from EDX mapping in Figure 3b further substantiate the uniform integration of PPy chains and MXene nanosheets.

## 4. Working Mechanisms

**Figure 4:**
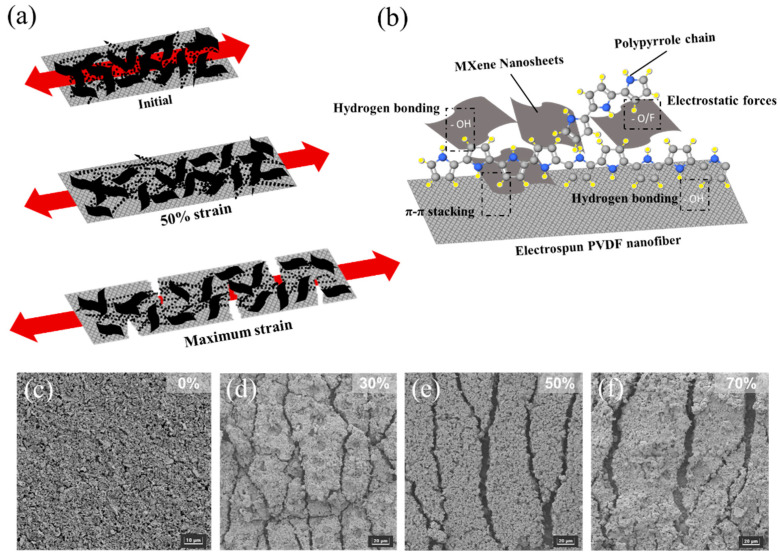
(**a**) Proposed sensing mechanism of Mxene/PPy electrospun PVDF sensor. (**b**) Illustration of chemical structure and interaction of MXene/PPy blending into the PVDF matrix to form an H-bonding. Morphology of the Ti_3_C_2_T_x_/PPy_0.4_/PVDF under tensile strain of (**c**) 0% (**d**) 30% (**e**) 50% (**f**) 70%.

The working mechanisms of the sensor were investigated by the morphological evolution of Ti_3_C_2_T_x_/PPy/PVDF under different applied tensile strains. Figure 4a demonstrates the schematic diagram of the initial stage till maximum strain is applied on the fabricated sensor. Figure 4b is the chemical structure and the scheme illustrating the chemical bond interactions between MXene, PPy and electrospun PVDF. With the introduction of PPy, strong bonds such as hydrogen bonding exist between the PPy chains, MXene nanosheets and PVDF nanofiber. Besides hydrogen bonding, H or F atoms (present in PVDF polymer and MXene surface functionalized group) has electrostatic attractions which provides stability to the layer structure [17]. As shown in Figure 4c, the Ti_3_C_2_T_x_/PPy/PVDF under a relaxed state exhibits randomly distributed MXene nanosheets on PVDF fibers, interconnected as a network structure. 

Before stretching, the conductive layer surface is smooth with an integrated conductive network. Tensile strain was calculated as (L − L_0_)/L_0_, where L and L_0_ are deformed and original lengths, respectively. Figure 4d–f shows the morphology of the Ti_3_C_2_T_x_/PPy_0.4_/PVDF under different elongation of strain. During tensile strain loading, the film is stretched along with the conductive network. At small and medium strains, the MXene and polypyrrole layer begins to break and slide from the PVDF fiber. Several microcracks appear on the surface of the MXene nanosheets layer indicating damage to the conductive network. There is still part of the polypyrrole network distributed randomly within the layer acting as bridges to connect the MXene nanosheets. With increasing tensile strain, the gaps between microcracks on the surface of MXene coating become wider. The microcracks are the mechanically weak point of the conductive network. The increase in microcracks and the propagation of cracks formation contributed to the variation of resistance across the conductive network. Eventually, the microcracks further widen during tensile strain, and the conductive network is destroyed. As a result, the electrical resistance of the conductive film increased sharply, similar to previously reported results [26,27,28] as shown in Table 1. The sensitivity of the sensor is developed from both polypyrrole network and MXene nanosheets in the conductive network layer. The network structure of PVDF fiber thus provides a buffer for strain loading for large deformation, thus preserving the conductive network layer from peeling or disruption. After a repeated stretch-release cycle, the conductive MXene layer is still intact with the PVDF fiber substrate which can be attributed to good interfacial stability between electrospun PVDF fiber and the MXene sheets [29]. The stable interface may be due to the strong H-bonding interaction of the MXene surface functional group with PVDF fiber [30].

## 5. Electromechanical Characterization and Sensing Demonstrations

**Figure 5:**
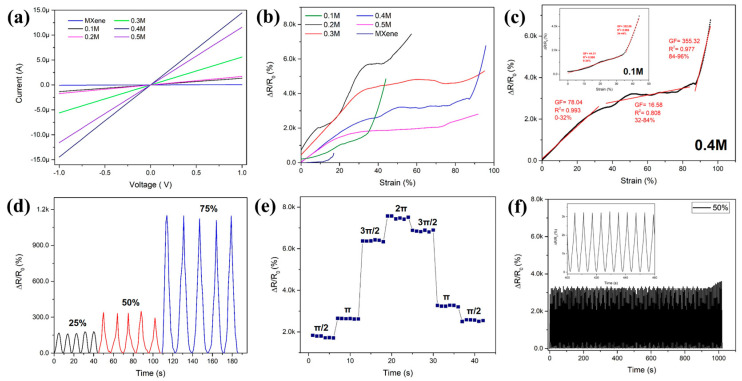
Electromechanical properties of the Ti_3_C_2_T_x_/PPy coated electrospun PVDF strain sensor. (**a**) Comparison of the variation of resistance change under quasi-static test for various FeCl_3_ concentrations used to fabricate the sensor. (**b**) The relative resistance changes of various concentration of FeCl_3_ fabricated sensor. (**c**) The relative resistance changes of 0.4M FeCl_3_ fabricated sensor (**d**) Relative resistance change of the sensor under stretch–release cycles at maximum strain of 25%, 50%, and 75%, respectively. (**e**) Torsion performance of the sensor with relative resistance changes under different torsion degrees. (**f**) Stability test under the strain of 50% for 1000 s.

The performance of the strain sensor based on Ti_3_C_2_T_x_/Polypyrrole electrospun PVDF was investigated. The fabricated Ti_3_C_2_T_x_/PPy/PVDF substrate for strain sensing can be tuned by adjusting the concentration of FeCl_3_, which is the initiator used to deposit polypyrrole and MXene nanosheets. Figure 5a shows the I–V curves are straight and smooth lines. As the concentration of FeCl_3_ increase, the resistance of the Ti_3_C_2_T_x_/PPy/PVDF substrate reduce. When the concentration of FeCl_3_ reaches 0.5M, the resistance increases. This may be due to the breakage of Ti_3_C_2_T_x_ nanosheets and polypyrrole strain. Thick deposition layers of conductive filler may result in peeling the conductive filler layer from the nanofiber structures.

Figure 5b shows the relative resistance variation-strain curve (ΔR/R_0-_ε) of the Ti_3_C_2_T_x_/PPy/PVDF. Generally, the relative resistance (ΔR/R_0_) where ΔR represents (R-R_0_), R and R_0_ indicate resistance under different strain and initial resistance, respectively. ε represents the strain. This gauge factor (GF) was used to evaluate the sensitivity of the strain sensor represented by the slope of the curve ΔR/R_0_-ε. From Figure 5c insets, the strain sensor based on Ti_3_C_2_T_x_/PPy_0.1_/PVDF was divided into two stages. During the strain range of 0–34%, the GF was quantified as 44.31 while the GF increased in the range of 34–44% to 352.86. The sensitivity of the sensor increases with low and smaller strain range. This could be due to the disconnection of microcrack junctions during tensile strain. When strain range increases, more microcracks appear, increasing crack-gap and crack density which can be identified with the distinct increase of GF and rapid decrease in electrical conductance. Therefore, the Ti_3_C_2_T_x_/PPy/PVDF composite-based strain sensor exceeds the performance of a conventional metal foil-based sensor with GF ≈ 2, strain range of 5% [31]. Table 1 shows the GF of reported sensors for comparison. As the concentration of FeCl_3_ increases, more deposition and compact network of the conductive fiber network. Thus, Ti_3_C_2_T_x_/PPy_0.4_/PVDF shows a higher GF of 78.04 with a range of 0–32%, shown in Figure 5c. At a higher strain range of 84–96%, the sensor exhibited a GF of 355.32. Figure 5d shows the frequency response at strain ranges of 25%, 50%, and 75%. The corresponding response and recovery of each measured signal share a similar shape and height, indicating the sensor has a stable response to the applied cyclic tensile test. The final resistance for strain range 25%, 50%, and 75% is 165 Ω, 332 Ω, and 1.1 kΩ, respectively. Furthermore, upon release of the strain sensor, the sensor reverts back to its initial resistance without noticeable hysteresis. In addition to the tensile strain sensing, the Ti_3_C_2_T_x_/PPy/PVDF sensor also demonstrate torsional motion. Figure 5e shows the relative resistance change during torsion deformation of the sensor. As the twist angle increase from 0, π/2, π, 3π/2, and 2π radians, the corresponding resistance changes from 0 to 2 kΩ with a subsequent increment of 2 kΩ until the sensor is fully twisted at 360°. As the deformation is released, the resistance change recovers and returns to the initial state with a difference of 745 Ω. Figure 5f demonstrates that during 1000 s cyclic stability test, the sensor undergoes a stretching-releasing cycle and shows a stable resistance response with good reversibility due to the strong interfacial interaction of the conductive layer.

**Figure 6:**
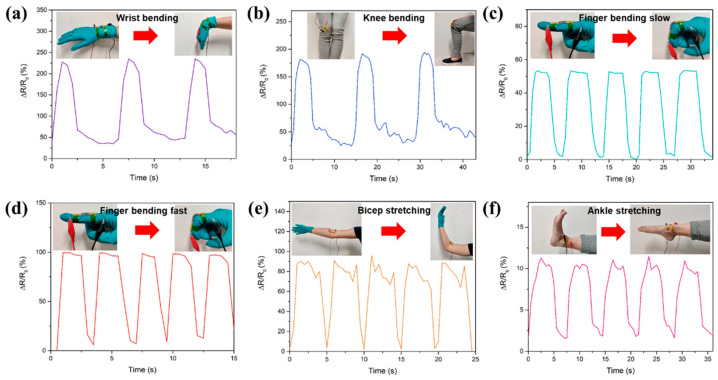
Application of the sensor in monitoring human body movement. (**a**) wrist bending (**b**) knee bending (**c**) finger bending at a slow pace (**d**) finger bending at a fast pace (**e**) bicep stretching (**f**) ankle stretching.

The developed Ti_3_C_2_T_x_/PPy/PVDF film is demonstrated as a wearable strain sensor, whereby the real-time electrical signal response in monitoring different human body motions is shown in Figure 6. The output signal shows a clear resistance change when the wrist is bend as shown in Figure 6a. Stable signal response is observed as the bending cycle with a resistance change of 22% is repeated. Figure 6b shows knee bending, represented by the squat and rising. The ER of the sensor increased rapidly by 17%, and in subsequent repeated action, the sensor was able to detect and maintain resistance change to the initial level. Besides large stretch and bending motions, small stretch human body parts are investigated. Finger motion monitoring includes actions of finger bending and straightening. The stable waveform is observed in Figure 6c,d, where bending cycles are repeated with 50% and 100% resistance changes, respectively. The difference is due to the frequency of finger bending. Essentially, the output signals correspond with the movements in which the resistance change is repeated in a similar amplitude. As shown in Figure 6e,f, human joint motions are monitored and investigated. Both biceps and ankle stretching demonstrate reproducible stretching cycles. During the initial stretch, ER value increases rapidly and changes until the sensor recovers to its original state. The bending and recovery test results indicate the sensor has reliability and durability in the application for human motion monitoring. The proof-of-concept in this demonstration of the Ti_3_C_2_T_x_/PPy/PVDF sensor suggests the potential of the fabricated sensor to be employed in the area of health care diagnostic and physical management. 

## 6. Conclusions

In summary, the Ti_3_C_2_T_x_ based strain sensor is successfully fabricated with a strategic configuration of Ti_3_C_2_T_x_ doped with Polypyrrole on flexible electrospun PVDF fiber. The fabricated composite of the substrate with microcracks exhibits a balance of sensitivity and sensing range that could be suitable for multiscale sensing. The hybrid structure of electrospun fiber as a foundation provides a percolation network for conductive filler network formation, which endows the sensor with excellent sensitivity. The fabricated sensor shows sensing performance characterized by sensitivity with a gauge factor of 78–355.32 with a sensing range of 0–100%. Besides its sensing properties, the sensor can be adopted for real-time human motion detection, thus providing a feasible approach for wearable strain sensors for healthcare monitoring.

## Figures and Tables

**Table 1:** Comparison of different material fabricated strain sensors.

Materials & Structure	Gauge Factor	Sensing Range (%)	Ref.
Conventional metal foil	2	0–5	[31]
MXene/polyimide film	46–180.1	0–2.13	[32]
MXene/air-laid paper	1–2.58	10–90	
Composite yarn doped PPy	51.2	0–40	[33]
AgNW–Ecoflex	0.7	0–50	[34]
Polypyrrole (PPy)/PU	1.3	0–40	[16]
Graphene/PDMS	151	0–5	[35]
Carbon Black/PDMS	12	0–30	[36]
MXene/PPy/Electrospun PVDF	44.31–355.32	0–100	This work
